# Supplementary material for: Chromothripsis during telomere crisis is independent of NHEJ, and consistent with a replicative origin
Source: Genome Res. 2019 May;29(5):737–49. doi: 10.1101/gr.240705.118 (PMC6499312; doi:10.1101/gr.240705.118)
Supplement: Supplemental Material [file supp_gr.240705.118_Supplemental_file_1.zip › contigs/annotated_contigs/DB113/contig.3.DB113_length_656_mean_cov_12.8460365854.docx]

**DB113_length_656_mean_cov_12.8460365854**

```
TATTAATGATGAAAGTTCCTTACTTTTC|TTTCTGTTTTTTTTTTTT|TTTTGAGATGGAGTCTTGCTCTGTCGCCCAGGCTGGAGTGC
 >chrUn_gl000219:54476-54504 - E=4e-05 p=8e-03  >chr6:159207113-159207409 - E=2e-163
AGTGGCACGATCTTGGCTCACTGCAACCTCCACCTCCTGGGTTCAAGTGATTCTCCTGCCTCAGCCTCCCAAGTAGCTGGGACTACAGC

TGCATGCCATCATGCCCGGCTAATTTTTTTGTATTTTTAGTAGAGATGGGGTTTCACCATGTTGGTCAGGCTAGTCTCGAACTCCTGAC

CTCGTGGTCCGCCCACCTCAGCCTCCCAAAGTGCTGGGATTACAGTCGTGAGCCACCATGCCTGGCCTGTTTTTTTG|CAGAGC|CCAC
                                                                                    >ch
TGTAGACAATTAACCTCATTGAGAGAAACGGGAGGATTTGGGAGTGAGGGGCAGGGGGCATTCTGAGCTGGGCTTTGTGGGGAGAGGCA
r6:159481294-159481602 - E=1e-173
GGATTGGGCCTTTGGAGAAGAGTGGAAAGGACATTTCAGTAGAGGGACAAAAGGGATGAAGGCCCAGAGATGCAAAGCACCCTTCACCC

ACAGAAGGCAGCAGGCATAGCAGAGGAGGGCTGAACGGGATTTGGTATCTGCCCGTCCCTTCCTTTCTGCTCCATAAGCTACTCCCATC

ATCCCCACTGTCATGGCCATGACCGACACTGAGTTAA
```
